# Supplementary material for: Prediction of sub-pyramid texturing as the next step towards high efficiency silicon heterojunction solar cells
Source: Nat Commun. 2023 Jun 16;14:3596. doi: 10.1038/s41467-023-39342-3 (PMC10275866; doi:10.1038/s41467-023-39342-3)
Supplement: Supplementary file 1 — Supplementary Information [file 41467_2023_39342_MOESM1_ESM.pdf]

Supporting Information

Prediction of sub-pyramid texturing as the next step towards high efficiency silicon heterojunction solar cells

Feihong Chu[1†], Xianlin Qu[2†], Yongcai He[1,3], Wenling Li[1], Xiaoqing Chen[1], Zilong Zheng[1*], Miao Yang[3], Xiaoning Ru[3], Fuguo Peng[3], Minghao Qu[3], Kun Zheng[1*], Xixiang Xu[3*], Hui Yan[1], Yongzhe Zhang[1*]

1. Faculty of Materials and Manufacturing, Faculty of Information Technology, Beijing University of Technology, Beijing, China
2. Center for Microscopy and Analysis, Nanjing University of Aeronautics and Astronautics, Nanjing, China
3. LONGi Central R&D Institute, Xi'an, China

†These authors contributed equally to this work.

*Correspondence Author (Email: zilong.zheng@bjut.edu.cn; kunzheng@bjut.edu.cn; xuxixiang@longi.com; yzzhang@bjut.edu.cn)

Supplementary Note 1: The anisotropy of c-Si epitaxy and nanotwin on (100), (011), (111) plane.

Considering the surface dangling bonds and atomic arrangement of low-index crystal plane, 3-atom nucleation is needed for further epitaxial growth on (111) plane, and 2-atom or 1-atom nucleation is needed on the (011) or (100) plane, respectively.[1] The number of atoms needed for nucleation has a strong influence on the epitaxy rate, therefore, the orientation dependent c-Si epitaxy rates are $v_{c(100)} > v_{c(011)} > v_{c(111)}$. Considering high epitaxy rate of (100) and (011) plane, the lateral spread of epitaxy will produce (111) steps, which leads to formation rough surface. In addition, the growth of (111) facets easily introduce stacking faults, because 3-atom nucleation on (111) facets can assume a twin configuration with coherent Σ3 (111) twin boundary. Coherent boundary is a boundary where there is complete continuity of atoms across it, i.e. there is one to one correspondence of atoms at the boundary/interface. Therefore, the coherent Σ3 (111) twin boundary usually forms after nucleation in the (111) plane, rather than high-energy non-coherent twin boundary.[2]

Supplementary Note 2: The effect of the H atom on the pure (111) and hybrid (111)/(011) plane

In order to describe the effect of the H atom on the pure (111) and hybrid (111)/(011) plane, we performed all atom molecular dynamics simulations for c-Si/a-Si:H interfacial morphology with 10% hydrogen content at high-temperature (1000 K) for 300 ns, considering the time-consuming MD simulations. The growth rate of epitaxial c-Si and twin-Si at the c-Si/a-Si:H interface were obtained, as shown in Fig. S9. The c-Si epitaxy rate (1.0×10^{14} $cm^{-2}\cdot\mu s^{-1}$) with 1000 K at c-Si/a-Si:H interface was similar to that (1.4×10^{14} $cm^{-2}\cdot\mu s^{-1}$) of c-Si/a-Si interface with 500 K. The H atoms contribute to reduce the epitaxial c-Si rate, but there is not significant difference.

On the other hand, at c-Si/a-Si:H interface, comparing with the c-Si epitaxial rate (1.0×10^{14} $cm^{-2}\cdot\mu s^{-1}$) on pure (111) plane, the hybrid (111)/(011) plane presented lower epitaxial c-Si rate (0.8×10^{14} $cm^{-2}\cdot\mu s^{-1}$), which was the consistent with c-Si/a-Si interface. The additional H atoms with 10% content did not presented significant influence on the epitaxial c-Si on pure (111) or hybrid (111)/(011) plane. In addition, the twin-Si start

reducing with the rate of -2.1×10^{14} $cm^{-2}\cdot\mu s^{-1}$ after 300 ns, and this phenomenon is consistent with c-Si/a-Si interface, which showed that the hybrid (111)/(011) plane inhibited the growth of twin-Si.

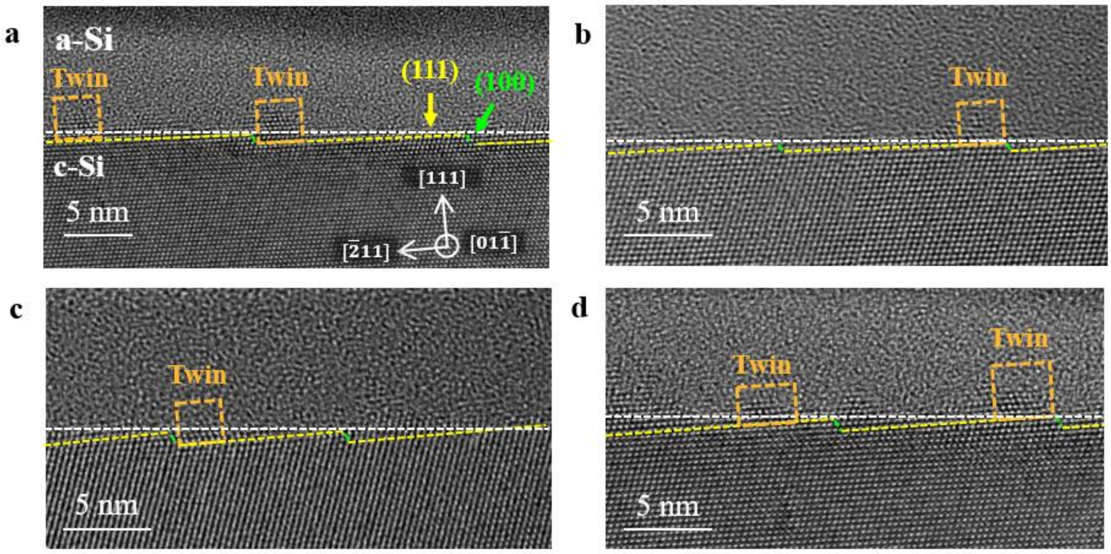

Supplementary Figure S1 | a-d Four HRTEM images of nanotwin distribution at hybrid $(111)_{0.9}/(100)_{0.1}$ planes. The yellow dashed line represents the (111) facet, the green dashed line represents the (100) facet, white dashed line represents c-Si surface and the orange rectangle represents nanotwin. The hybrid plane is composed of (111) and (100) facets. The nanotwins grown on the (111) facet and close to the (100) facet.

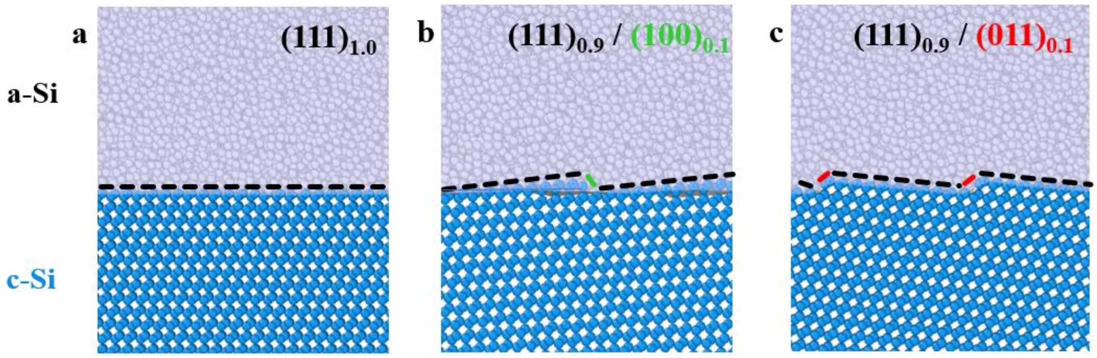

Supplementary Figure S2 | a-c The atomic models of c-Si/a-Si interface. The atomic models of pure (111) (**a**), hybrid $(111)_{0.9}/(100)_{0.1}$ (**b**) and hybrid $(111)_{0.9}/(011)_{0.1}$ (**c**) planes. The c-Si and a-Si atoms in the models are marked in blue and gray, respectively. The black dashed line represents the (111) facet, the green dashed line represents the (100) facet and the red dashed line represents the (011) facet.

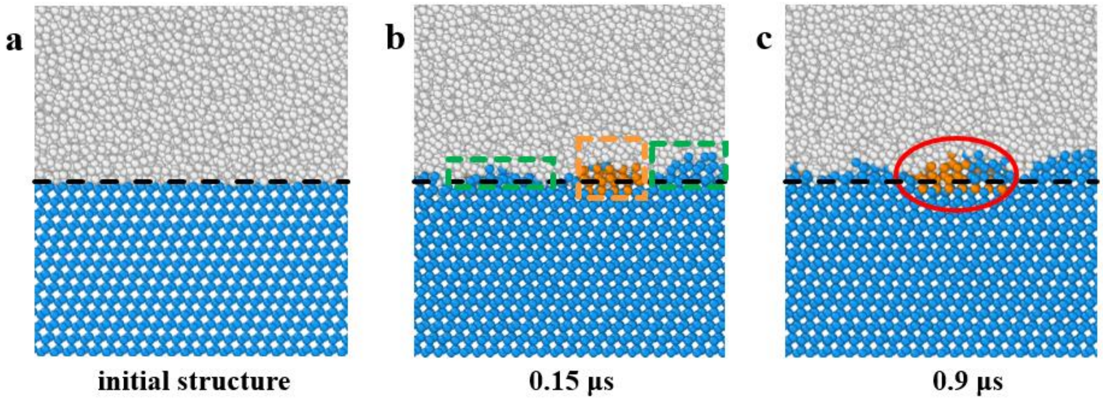

Supplementary Figure S3 | a-c, Interfacial morphological on pure $(111)_{1.0}$ plane at 0 μs (**a**), 0.15 μs (**b**) and 0.9 μs (**c**). The c-Si, a-Si and twin-Si atoms are marked in blue, gray and orange, respectively. The black dashed line represents the (111) plane, the green dashed rectangle represents epitaxial c-Si, the orange dashed rectangle represents nanotwin and the red oval represents embedded nanotwin.

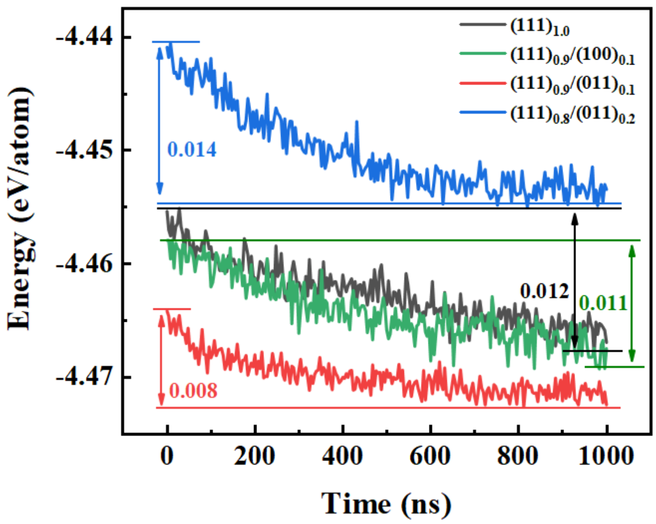

Supplementary Figure S4 | The average energy per atom at c-Si/a-Si interface. The energy is proportional to the driving force for the conversion of a-Si atoms to c-Si atoms. Compared with pure $(111)_{1.0}$ plane, the hybrid $(111)_{0.9}/(011)_{0.1}$ plane has lower driving force for the conversion of a-Si atoms to c-Si atoms, but increasing the (011) component (x) of the hybrid plane to hybrid $(111)_{0.8}/(011)_{0.2}$ results in enhanced driving force. The difference in energy at the same time indicates the number of a-Si atoms converted to c-Si atoms. Large driving forces lead to fast conversion of a-Si atoms to c-Si atoms, resulting in enhanced epitaxy.

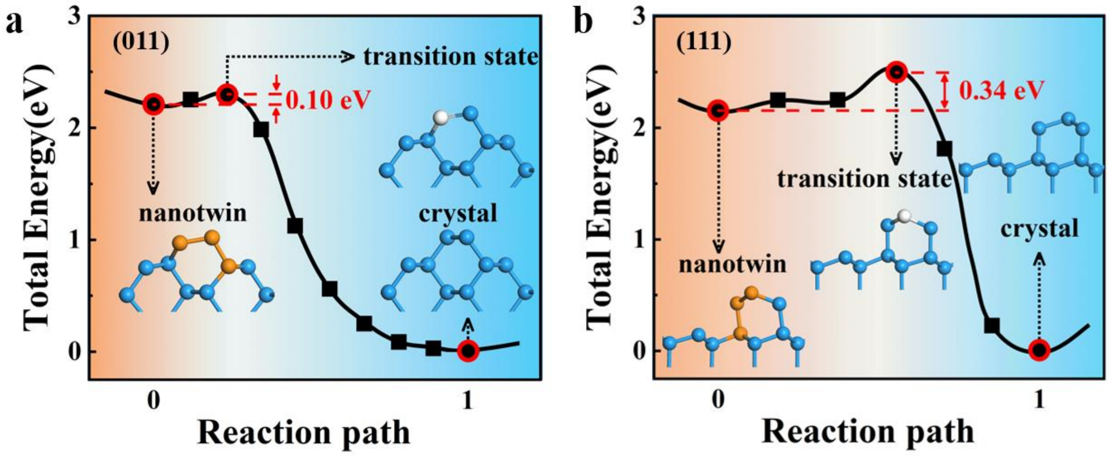

Supplementary Figure S5 | a,b The transition states of twin-Si atoms converted to c-Si atoms on (100) (**a**) and (111) (**b**) planes. Structure containing twin-Si atoms are about 2 eV higher in energy than that of c-Si atoms, indicating that the formation of epitaxial c-Si is more stable than the nanotwin. The transition of twin-Si converted to c-Si on the (011) plane requires a potential barrier of 0.1 eV to be overcome, while energy barrier on (111) surface is 0.3 eV. The potential barrier of the (011) plane is significantly smaller than that of the (111) plane, which indicates that the twin-Si on the (011) plane is unstable compared to that on the (111) plane, and nanotwin is easier to convert to c-Si epitaxy under temperature of 500 K.

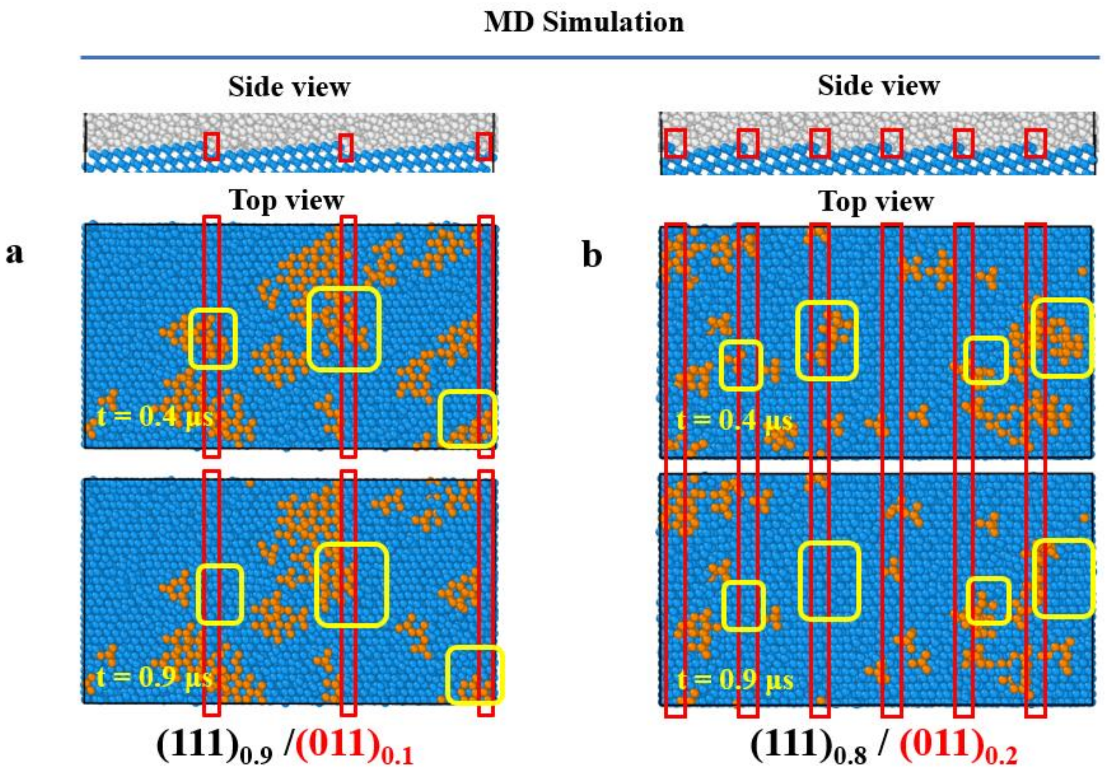

Supplementary Figure S6 | a,b Nanotwin distribution at interface for hybrid $(111)_{0.9}/(011)_{0.1}$ (**a**) and $(111)_{0.8}/(011)_{0.2}$ (**b**) plane. The side view (**top panel**) of c-Si/a-Si interface of initial hybrid plane, and top view of c-Si/a-Si interface of hybrid plane, following 0.4 μs (**middle panel**) and 0.9 μs (**bottom panel**) low-temperature (500 K) molecular dynamic simulations. The red rectangle represents (011) facet. The yellow rectangle represents the disappearance of nanotwins. The nanotwin disappear mainly on or close to the (011) plane. Since the twin-Si disappearance rate is proportional to the (011) component (x), therefore, the twin-Si disappearance rate of hybrid $(111)_{0.8}/(011)_{0.2}$ plane is faster than that of $(111)_{0.9}/(011)_{0.1}$ plane.

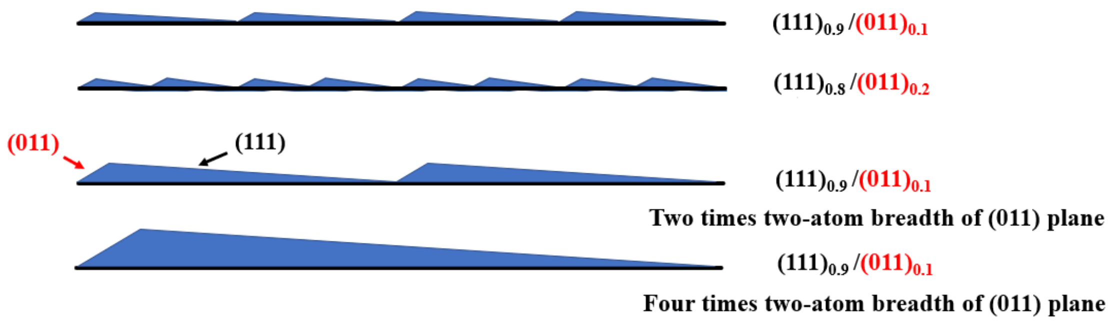

Supplementary Figure S7 | The influence of the component and breadth of (011) plane on the atomic step distribution. When the breadth of (011) facet is kept consistent, increasing the (011) component leads to an increase in step density. Keeping the (011) component, increasing the breadth of (011) plane leads to a decrease in step density.

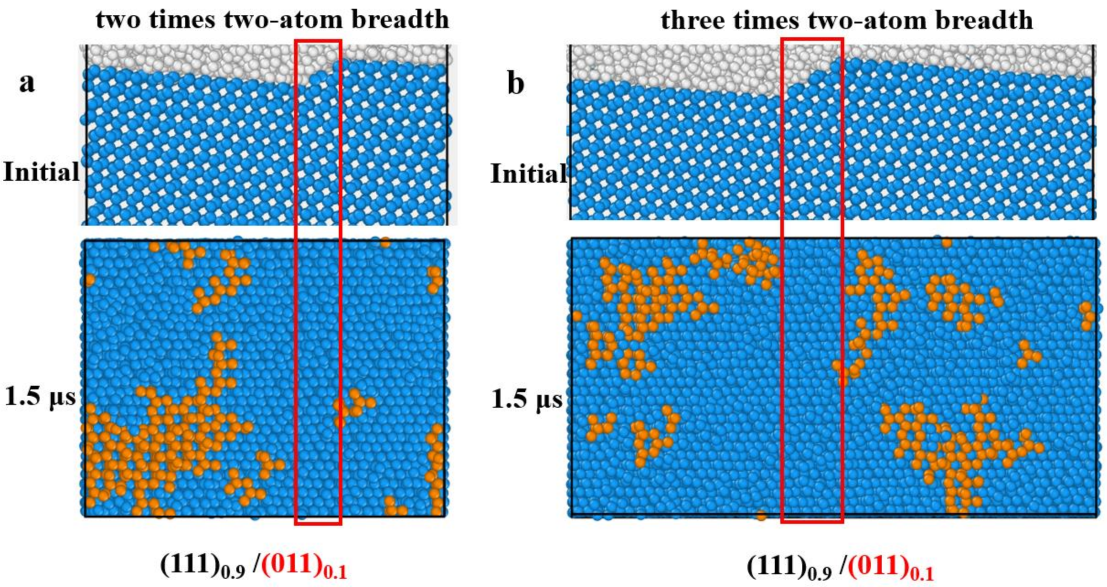

Supplementary Figure S8 | a,b Nanotwin distribution on interface for $(111)_{0.9}/(011)_{0.1}$ plane with two (**a**) and three (**b**) times breadth of (011) facet. The side view (**top panel**) of c-Si/a-Si interface of initial hybrid plane, top view (**bottom panel**) of c-Si/a-Si interface of hybrid plane after heating at 500 K for 1.5 μs. The red rectangle represents (011) facet.

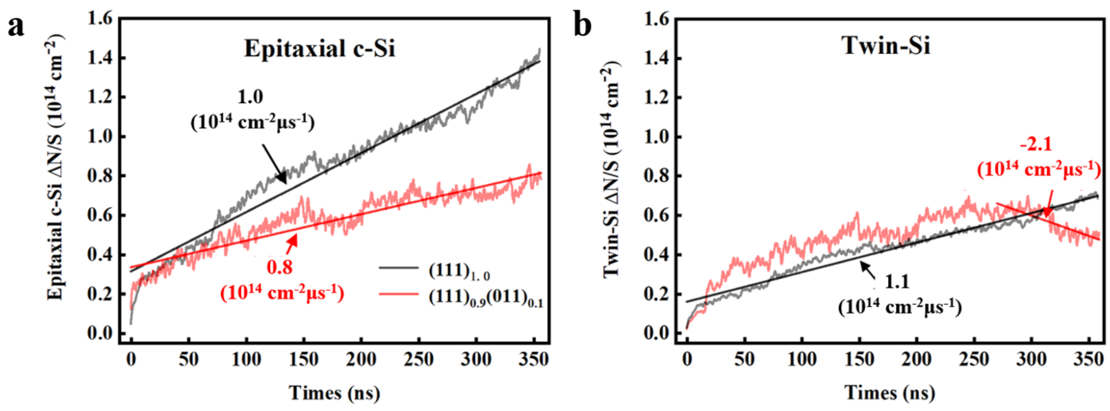

Supplementary Figure S9 | Molecular dynamic simulations at c-Si/a-Si:H interface on pure (111) c-Si plane and hybrid $(111)_{0.9}/(011)_{0.1}$ c-Si plane. The generation of both (a) epitaxial c-Si and (b) twin-Si atoms as a function of time.

Supplementary References:

1. Ueno T, Showya T, Ohdomari I. Atomic scale structure of microtwins in single crystal Si grown by lateral solid phase epitaxy. *J. Appl. Phys.* **69,** 808-811 (1991).
2. Cahn RW. Twinned crystals. *Adv. Phys.* **3,** 363-445 (1954).
