# Supplementary material for: Prediction of sub-pyramid texturing as the next step towards high efficiency silicon heterojunction solar cells
Source: Nat Commun. 2023 Jun 16;14:3596. doi: 10.1038/s41467-023-39342-3 (PMC10275866; doi:10.1038/s41467-023-39342-3)
Supplement: Supplementary file 3 — Solar Cells Reporting Summary [file 41467_2023_39342_MOESM3_ESM.pdf]

Corresponding author(s): Zilong Zheng

Solar Cells Reporting Summary

Nature Research wishes to improve the reproducibility of the work that we publish. This form is intended for publication with all accepted papers reporting the characterization of photovoltaic devices and provides structure for consistency and transparency in reporting. Some list items might not apply to an individual manuscript, but all fields must be completed for clarity.

For further information on Nature Research policies, including our data availability policy, see Authors & Referees.

Experimental design

Please check: are the following details reported in the manuscript?

1. Dimensions

Area of the tested solar cells

☒ Yes ☐ No

The relevant area of the tested solar cells s 244.5 cm2, and is located in the first paragraph of the discussion.

Method used to determine the device area

☒ Yes ☐ No

We used a standard commercial silicon wafer(M2), and the relevant method is located in the first paragraph of the discussion.

2. Current-voltage characterization

Current density-voltage (J-V) plots in both forward and backward direction

☐ Yes ☒ No

We mainly focused on the interface morphology of SHJ solar cell, therefore, current-voltage characterization was not shown. If current-voltage characterization is needed, we can provide the relevant data in Support Information.

Voltage scan conditions
For instance: scan direction, speed, dwell times

☐ Yes ☒ No

We tested SHJ solar cell by standard test method.

Test environment
For instance: characterization temperature, in air or in glove box

☐ Yes ☒ No

We tested SHJ solar cell by standard test method.

Protocol for preconditioning of the device before its characterization

☐ Yes ☒ No

We tested SHJ solar cell by standard test method.

Stability of the J-V characteristic
Verified with time evolution of the maximum power point or with the photocurrent at maximum power point; see ref. 7 for details.

☐ Yes ☒ No

The efficiency of SHJ solar cell did not degradation significantly with the test time.

3. Hysteresis or any other unusual behaviour

Description of the unusual behaviour observed during the characterization

☐ Yes ☒ No

In our test, SHJ solar did not show hysteresis and any other unusual behavior.

Related experimental data

☐ Yes ☒ No

SHJ solar did not show hysteresis and any other unusual behavior.

4. Efficiency

External quantum efficiency (EQE) or incident photons to current efficiency (IPCE)

☐ Yes ☒ No

We mainly focus on the interface morphology of SHJ solar cell, therefore, the IPCE was not provided.

A comparison between the integrated response under the standard reference spectrum and the response measure under the simulator

☐ Yes ☒ No

The response of our single junction devices is almost same between reference spectrum and simulator due to the 3A level light source with YSS-180S-IVT(VS-0851A) simulator. In that case, we did not compare the integrated response under reference spectrum and simulator.

For tandem solar cells, the bias illumination and bias voltage used for each subcell

☐ Yes ☒ No

Our SHJ solar cells were single junction, not tandem solar cells.

5. Calibration

Light source and reference cell or sensor used for the characterization

☐ Yes ☒ No

Our light source is YSS-180S-IVT(VS-0851A). We mainly focus on the interface morphology of SHJ solar cell, thus light source was not provided in our manuscript.

Confirmation that the reference cell was calibrated and certified

☐ Yes
☒ No

Our reference cell was certified by IFSH. We mainly focus on the interface morphology of SHJ solar cell, thus we did not provided in our manuscript.

Calculation of spectral mismatch between the reference cell and the devices under test

☐ Yes
☒ No

Our reference cell was single junction without spectral mismatch.

6. Mask/aperture

Size of the mask/aperture used during testing

☐ Yes
☒ No

Our solar cell substrate was the standard commercial silicon wafer sizes M2. Thus we did not use the mask.

Variation of the measured short-circuit current density with the mask/aperture area

☐ Yes
☒ No

Our solar cell subtract was the standard commercial silicon wafer sizes M2. Thus we did not use the mask.

7. Performance certification

Identity of the independent certification laboratory that confirmed the photovoltaic performance

☐ Yes
☒ No

Our reference cell was certified by IFSH. We mainly focus on the interface morphology of SHJ solar cell, thus we did not provided in our manuscript.

A copy of any certificate(s)
Provide in Supplementary Information

☐ Yes
☒ No

We mainly focus on the interface morphology of SHJ solar cell, thus we did not provided in our manuscript.

8. Statistics

Number of solar cells tested

☐ Yes
☒ No

We have tested five devices, and they had almost same efficiency.

Statistical analysis of the device performance

☐ Yes
☒ No

Our previous work has published the statistical analysis of solar cells performance, and we used one of devices to investigate interface morphology. The detail results can be found in doi:10.1038/s41560-020-00768-4.

9. Long-term stability analysis

Type of analysis, bias conditions and environmental conditions
For instance: illumination type, temperature, atmosphere humidity, encapsulation method, preconditioning temperature

☐ Yes
☒ No

The performance of SHJ solar cell is quiet stable, thus we not tracked long-term stability.
